# Fibrocystin Is Essential to Cellular Control of Adhesion and Epithelial Morphogenesis

**DOI:** 10.3390/ijms21145140

**Published:** 2020-07-20

**Authors:** Wolfgang H. Ziegler, Birga Soetje, Lisa P. Marten, Jana Wiese, Mithila Burute, Dieter Haffner

**Affiliations:** 1 Department of Pediatric Kidney, Liver and Metabolic Diseases, Hannover Medical School, 30625 Hannover, Germany; b.soetje@gmx.de (B.S.); lisapilar.marten@web.de (L.P.M.); jana.wiese92@gmail.com (J.W.); haffner.dieter@mh-hannover.de (D.H.); 2 Université Paris Diderot, CEA, INSERM, Hôpital Saint Louis, Institut Universitaire d’Hematologie, UMRS1160, CytoMorpho Lab, 75010 Paris, France; mithila.pune@gmail.com

**Keywords:** polycystic kidney disease, fibrocystin, adhesion signaling, epithelial polarity, lumen formation

## Abstract

Mutations of the *Pkhd1* gene cause autosomal recessive polycystic kidney disease (ARPKD). *Pkhd1* encodes fibrocystin/polyductin (FPC), a ciliary type I membrane protein of largely unknown function, suggested to affect adhesion signaling of cells. Contributions of epithelial cell adhesion and contractility to the disease process are elusive. Here, we link loss of FPC to defective epithelial morphogenesis in 3D cell culture and altered cell contact formation. We study *Pkhd1*-silenced Madin-Darby Canine Kidney II (MDCKII) cells using an epithelial morphogenesis assay based on micropatterned glass coverslips. The assay allows analysis of cell adhesion, polarity and lumen formation of epithelial spheroids. *Pkhd1* silencing critically affects the initial phase of the morphogenesis assay, leading to a reduction of correctly polarized spheroids by two thirds. Defects are characterized by altered cell adhesion and centrosome positioning of FPC-deficient cells in their 1-/2-cell stages. When myosin II inhibitor is applied to reduce cellular tension during the critical early phase of the assay, *Pkhd1* silencing no longer inhibits formation of correctly polarized epithelia. We propose that altered sensing and cell interaction of FPC-deficient epithelial cells promote progressive epithelial defects in ARPKD.

## 1. Introduction

Ciliopathies are rare (mostly) monogenetic diseases (or syndromes) caused by mutation i.e., loss of function, of proteins of the cilia-centrosome complex [1]. The range of inherited traits regularly comprises defects of epithelial morphogenesis or loss of epithelial tissue homeostasis, leading to epithelial dysplasia and/or cyst formation in kidney and less frequently liver and further organs, and is often accompanied by tissue fibrosis. Loss of epithelial homeostasis (regeneration) was suggested to associate with enhanced cell proliferation, defective (control of) epithelial polarity i.e., mislocalization of receptors and ion channels, and aberrant expression of embryonal subtypes of membrane proteins [2,3]. Autosomal recessive polycystic kidney disease (ARPKD), a ciliopathy and main cause of endstage renal disease (ESRD) in children, originates from mutagenesis of the polycystic kidney and hepatic disease 1 (PKHD1) gene and loss of function of the encoded protein fibrocystin/polyductin (FPC) [4,5]. So far, only symptomatic treatment of ARPKD is available. FPC is a type I membrane protein of 4074 amino acids with a small cytoplasmic domain, which can be released by Notch-like processing. Furthermore, the protein FPC localizes to cilia of collecting and bile duct epithelia of kidney and liver, respectively, and in proliferating cells in culture to centrosomes and the mitotic spindle [6,7,8]. FPC is expected to perform various functions in epithelial tissues, which are not understood mechanistically.

Throughout the bodies of multicellular organisms, epithelia form barriers that separate tissues from liquid- or gas-filled lumina. Epithelial function depends on establishment and maintenance of defined apicobasal polarity and control of cell(–cell) interactions in the epithelial plane that distribute forces and define permeability of the barrier [9,10,11]. Thus, characteristics of the epithelial sheet are mostly determined by three types of cell interactions all linked to the actin cytoskeleton, adherens junctions (AJ), tight junctions (TJ) and focal adhesions (FA). These interactions (i) connect different transmembrane receptors to actin cytoskeleton i.e., actomyosin-based cell contractility and (ii) establish apicobasal polarity, which is essential to selective permeability of the barrier [9]. Establishment of epithelial cell polarity and lumen formation are furthermore determined by positioning of centrosomes or basal bodies, their derivatives at the base of primary cilia, which define the orientation of microtubule-based intracellular trafficking of vesicles and membrane-bound protein [10,12,13].

In brief, the (i) distribution of cellular contractile elements (cell contractility) through connection of the actin cytoskeleton to different receptors and the (ii) positioning of centrosomes, the organization centers of microtubules (MTOCs), are pivotal to the function of epithelial sheets and influence maintenance of mechanical stability, force distribution and permeability of the barrier. Cellular control of both essential features can be assessed during epithelial cell spheroid formation. For Madin-Darby Canine Kidney II (MDCKII) cells, Rodríguez-Fraticelli and colleagues [13] developed an epithelial morphogenesis assay based on micropatterned glass coverslips with selective extracellular matrix (ECM)-coating so-called adhesion chips. The assay allows formation of epithelial spheroids (also termed cysts or acini) in matrigel-supplemented growth medium. Starting from a single cell, correctly polarized spheroids of 16–20 cells form with high incidence (>>80%) within 3 days of cell culture. Spheroid formation strictly depends on the balance of input signals through adhesion receptors and distribution of contractile forces [13]. Consequences of selectively modified input can be monitored by high-resolution imaging. Thus, the morphogenesis assay appears well-suited to reveal changes (or defects) in environmental sensing and adhesion signaling of MDCKII cells.

Here, we address consequences of a transient reduction of FPC protein on the capacity of collecting duct epithelial cells (MDCKII cells) to form correctly polarized, monolayered spheroids, i.e., to control initial steps of epithelial morphogenesis. The assay starting from a single cell specifically probes whether cell(–cell) interactions and balance of forces are altered in cells deficient of FPC function. In these conditions, MDCKII cells with insufficient FPC expression display a strongly compromised capacity to form correctly polarized epithelia, a result of altered behavior in their one-cell and two-cell stages. Upon treatment to reduce contractile forces during the critical, initial phase of spheroid formation, control of epithelial polarity is restored, confirming deficits of FPC-deficient cells in cellular control of cytoskeletal forces and adhesion signaling. Altered sensing and interaction of epithelial cells with their environment are suggested to lead to progressive defects in collecting and bile duct epithelia and eventually loss of organ function in ARPKD patients.

## 2. Results

**Figure 1:**
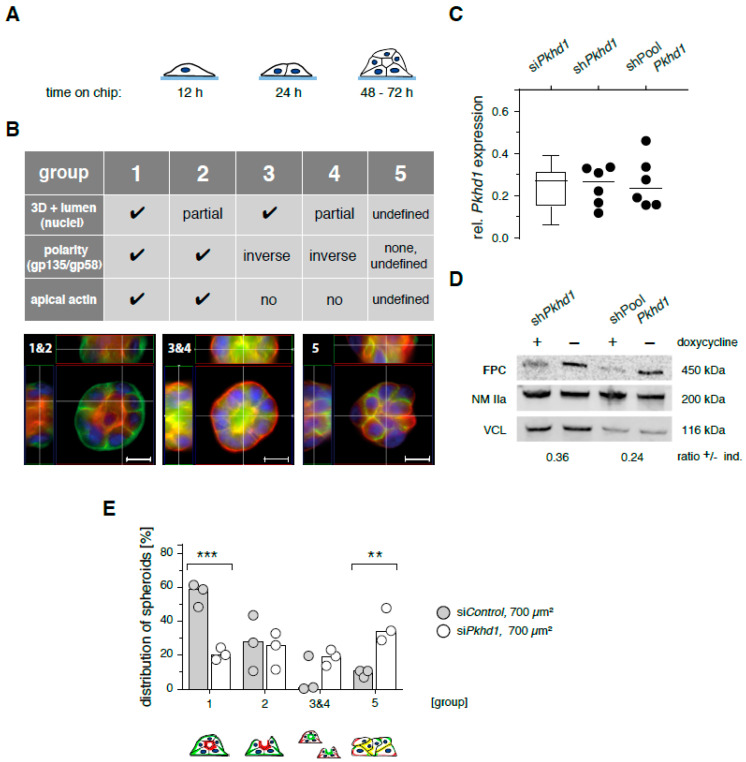
Induction and classification of epithelial spheroids; fibrocystin/polyductin (FPC)-deficient cells show defects in epithelial morphogenesis. (**A**) Madin-Darby Canine Kidney II (MDCKII) cells are seeded onto adhesion chips with extracellular matrix (ECM)-coated, disc-shaped micropatterns of 700 or 1600 µm^2^. Single cells give rise to spheroids of 16 to 20 cells within 3 days of culture. (**B**) Classification of spheroids; fixed cell clusters are stained for gp58 (basolateral, green), gp135/podocalyxin (apical, red), F-actin (not shown) and nuclei (DAPI, blue). Signals for podocalyxin and F-actin (phalloidin) correlate highly. Spheroids are analyzed based on blinded classification of z-stacks of 4-color fluorescence images. Size bars, 10 µm. (**C**) To control efficiency of knockdown, *Pkhd1* mRNA levels were determined by real-time polymerase chain reaction (PCR) using the ΔΔCT method relative to three reference genes. *Pkhd1* expression is given as ratio of levels from si*Pkhd1* to si*Control*-treated cells (*n* = 16 independent experiments, box plot with whiskers 5/95%) and sh*Pkhd1*/shPool*Pkhd1* to sh*Control*-treated cells (*n* = 6 independent experiments). Small interfering RNA (siRNA) constructs of si*Pkhd1*/sh*Pkhd1* and si*Control*/sh*Control* correspond, respectively. shPool*Pkhd1* combines four different small hairpin RNA (shRNA) sequences [7] against *Pkhd1* mRNA. (**D**) Reduced expression of FPC protein in MDCKII TetON-cells [17], 72 h after doxycycline-treatment-induced shRNA expression. Ratios give mean protein values of two independent experiments. Full-length immunoblots are provided in Supplementary Figure S2. (**E**) Spheroid formation by siRNA-treated MDCKII cells on 700 µm^2^ collagen-coated micropattern. Group characteristics are illustrated (below). (*n* = 3 independent experiments, median bar; >200 spheroids per condition; two-way analysis of variance (ANOVA)/Sidak’s, *p* < 0.01/0.001, **/***.)

*Epithelial morphogenesis by MDCKII cells is highly sensitive to adhesion signaling.* In cell culture, loss of FPC (function) from epithelial cells was proposed to associate with altered adhesion signaling and thus changes in environmental sensing and cell interactions [14]. To address consequences of defective cell behavior, we adapted the protocol of a spheroid assay based on micropattern adhesion chips [13]. Imposing a defined and selective environment, this assay allows analysis of cell adhesion and behavior from one-cell and two-cell stages to polarized epithelial cell spheroids (16–20 cells) with high spatial resolution and furthermore provides means of direct quantification (Figure 1A,B). Spheroid characteristics are determined based on z-stacks of four-colour fluorescence images providing information on (i) three dimensional (3D) structure and lumen formation (nuclei), (ii) position of apical (gp135/podocalyxin) and basolateral (gp58/β subunit of Na^+^/K^+^ ATPase) markers [15,16] and (iii) enrichment of contractile actin structures (apical actin). Cell clusters are classified into five groups corresponding to correctly polarized spheroids with liquid-filled lumen forming a complete or partial sphere (groups 1 and 2), inversely polarized spheroids with matrix-filled center and complete or partial sphere (groups 3 and 4) and unpolarized aggregates of cells (group 5), as illustrated in Figure 1B. Structures classified as groups 3 and 4 are rather rare events with inverted polarity, and group 5 indicates aggregates with no defined polarity and no lumen (or multiple lumina). These groups summarize all structures with defective cell interaction that do not give rise to a functional epithelium.

Function of the assay was confirmed as detailed in supplementary material, Figure S1. In the presence of low concentrations of matrigel in culture medium, adhesion of MDCKII cells to collagen-coated disc-shaped patterns of 700 µm^2^ (high cell confinement) provides a balanced mix of stimuli that induce formation of correct spheroids (groups 1 and 2) with high incidence of greater than 85%, Supplementary Figure S1A. In contrast, collagen-coated discs of 1600 µm^2^ (low cell confinement), which, due to their larger adhesive area do not mimic the spatial constraints imposed on cells in epithelial monolayers, generate 43% of groups 1 and 2 spheroids and more than 50% of cell aggregates. Furthermore, adhesion of cells to laminin, which induces only weak cell–ECM interactions, allows efficient formation of correct spheroids (groups 1 and 2) independent of the confinement imposed by disc-shaped patterns (700 or 1600 µm^2^). These data confirm that the spheroid assay is functional and operates in agreement with observations by Rodriguez-Fraticelli et al. [13]. Thus, in this in vitro assay, efficient formation of correctly polarized epithelium reflects balanced input signaling via receptor stimulation (culture medium), matrix-dependent adhesion signals and spatial confinement.

*Temporal loss of FPC function leads to defective spheroid formation.* Impaired FPC function is achieved by transient knockdown of *Pkhd1* expression using specific siRNA (si*Pkhd1*) or shRNA (sh*Pkhd1*) as validated and reported for MDCK cells by Zhang and colleagues [7]. Consistent with their results, we obtained transient reduction of *Pkhd1* mRNA levels down to 15–30% of control treatment. The reduction is observed 48 h after siRNA treatment and 48–72 h after induction of shRNA expression using doxycycline. We control effectiveness of siRNA/shRNA treatment using quantitative real-time PCR to determine mRNA levels of *Pkhd1* and three reference genes. Knockdown is similarly effective when using si*Pkhd1*, sh*Pkhd1* or the pool of four *Pkhd1*-specific shRNAs (shPool*Pkhd1*) that were described previously [7] (Figure 1C). In mouse cells, *Pkhd1* silencing was reported to alter PC2 expression [18]. Therefore, we confirmed that in our experimental setup, silencing did not affect Pkd2 mRNA levels (Figure S1B). To complete analysis of *Pkhd1* silencing, we also detected loss of the full-length FPC protein with an apparent molecular weight of 450 kDa in cells expressing sh*Pkhd1* and shPool*Pkhd1* with and without doxycycline induction (Figure 1D). Strong reduction of the low abundance protein is clearly detectable in extracts with very high total protein content. Without enhanced selection, which would not be compatible with transient knockdown, we expect effective siRNA/shRNA silencing in 80–90% of all treated cells, resulting in residual mRNA and protein signals of cell pools as detected. All analyses in this study depend on the use of or originate from single cells. Thus, consequences of transient *Pkhd1* silencing are underestimated due to approximately 20% of cells with no (or insufficient) effect of treatment.

In the spheroid assay, si*Pkhd1* treatment is timed to coincide maximal knockdown of FPC with cell seeding, one-cell and two-cell stages, while mRNA regains wildtype levels, when spheroids are ready for classification after three days (Supplementary Figure S1C). Applying this protocol on 700 µm^2^ collagen-coated micropatterns, we observed a reduction by 39% of group 1 spheroids to one-third of control values and a sharp 3-fold increase of cell aggregates (group 5) in si*Pkhd1* compared to si*Control*-treated cells (Figure 1E). Comparable reduction of correctly polarized spheroids (groups 1 and 2) was observed in cells silenced by doxycycline-induced expression of sh*Pkhd1* (or shPool*Pkhd1*) (Supplementary Figure S1D). Defective spheroid polarity indicates an imbalance of adhesion signals in MDCKII cells following acute reduction of FPC expression. When we tested seeding of cells immediately after si*Pkhd1* treatment, which leads to a transient loss of FPC protein in cells around completion of the spheroid assay, treatment was ineffective with no indication of a difference between silenced cells and controls. Use of si*Pkhd1* silencing is truly transient and shows a similar efficacy as compared to shRNA treatments, which in addition require doxycycline treatment, a further influence on the behavior of cells. Therefore, we performed subsequent analyses of FPC function in MDCKII cells treated for 48 h with si*Pkhd1* and controlled *Pkhd1* mRNA levels of cell pools to stay below 30% of control treatment.

**Figure 2:**
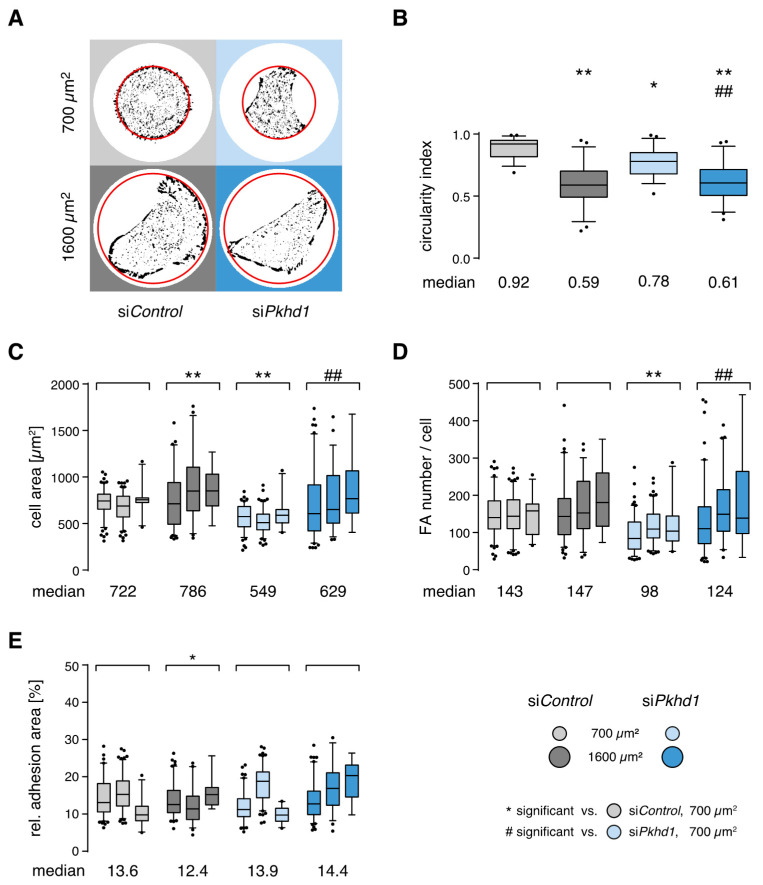
Effects of FPC deficiency and confinement on cell shape and adhesion sites. (**A**) Representative cell shape and adhesion sites of siRNA-treated cells. Four hours after seeding onto collagen-coated micropatterns, cells were fixed and stained for vinculin to mark cell–ECM adhesions. Red line indicates the outline of disk-shaped adhesion area; diameter size: 30 and 45 µm for 700 and 1600 µm^2^ pattern, respectively. (**B**) Cell geometry determined by relation of short to long cell axis (circularity index). All box plots with whiskers 5/95% (*n* = 50 cells per condition, two-way ANOVA/Tukey’s). (**C**–**E**) Spreading area, number of adhesion sites per cell and relative adhesion area extracted from fluorescence images of steady-state adhesion for FPC-deficient cells and controls in spheroid culture conditions. (*n* = 3 independent experiments, >250/170 cells per condition for 700/1600 µm^2^; two-way ANOVA/Tukey’s, *p* < 0.05/0.01, */** or **#**/**##**.)

*FPC-deficient cells show altered control of spreading and adhesion.* Results of the spheroid assay suggest a significant impact of FPC protein function on the initial steps of spheroid formation and by implication epithelial morphogenesis. Does this correspond to an altered control of cell shape and adhesion? To study this possibility, we collected quantitative adhesion parameters of MDCKII cells 4 h after seeding onto 700 or 1600 µm^2^ collagen-coated micropatterns, employing identical culture conditions as in the spheroid assay. After 4 h, cells are fully spread and adapted to cell-ECM adhesion (steady-state). Detection of FAs, by fluorescence staining of cytoskeletal adaptor proteins such as vinculin, allows extraction of number, size and area of adhesion sites, a semi-quantitative estimate of adhesion strength [19,20,21]. In addition, spreading area and cell shape as well as distribution of adhesion sites are extracted from images (Supplementary Figure S3A). Representative extracted adhesion sites (FAs) of si*Pkhd1* and si*Control*-treated cells on both sizes of collagen-coated micropatterns (700/1600 µm^2^) are shown in Figure 2A. On 700 µm^2^, the shape of si*Control* cells reflects adaptation to confinement by completely filling the available area, while adhesion to 1600 µm^2^ induces an elongated, polarized cell shape. This is observed also for both conditions by the circularity index measuring the ratio of the length axis to the perpendicular width of cells (illustration of measurement, Supplementary Figure S3B). In high confinement conditions, circularity of 0.92 for si*Control* cells indicates almost perfectly round shape (index of 1), whereas cells are more than 50% longer than wide with low lateral restriction (Figure 2A,B). Furthermore, cell spreading area of si*Control* cells is restricted on 700 µm^2^ and significantly increased by 8.9% on 1600 µm^2^. While the number of FAs remains unaltered, the increased spreading area leads to a decrease of the relative adhesion area, i.e., the ratio of total adhesion site area to cell area (Figure 2C–E).

In comparison, si*Pkhd1* cells lacking FPC protein are considerably smaller in size (24%) than si*Control* cells on 700 µm^2^, rarely filling the entire pattern, and show a moderately elongated shape (circularity of 0.78) (Figure 2A–C). To address possible effects of siRNA treatment, we estimated cell size/volume of si*Pkhd1* and si*Control* cells in suspension based on scattering parameters of FACS analysis and did not observe significant differences (Supplementary Figure S3C). Since si*Pkhd1* cells are less spread and also show a reduced number of FAs, the relative adhesion area (13.9%) is not altered, compared to si*Control* cells. In addition, release of confinement triggers a significant increase in cell spreading area (14.6%) and number of cell adhesions (26.5%), indicating that confinement also affects the control of adhesion sites in si*Pkhd1* cells (Figure 2C–E). There is no immediate explanation for the apparent incompetence of si*Pkhd1* cells to fill the 700 µm^2^ high confinement area.

**Figure 3:**
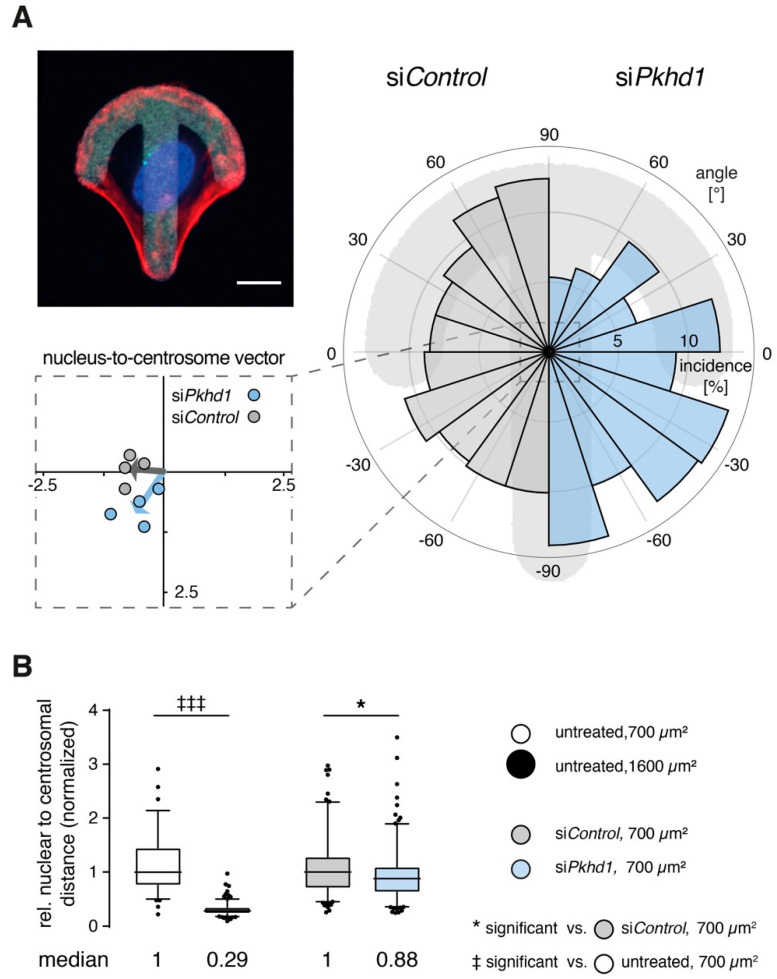
Defective positioning of centrosomes in 1 and 2-cell stages. (**A**) Adhesion of single cells on crossbow-shaped micropatterns (upper left) enforces positioning of centrosomes, in front of nucleus, corresponding to the polarized orientation of a migratory cell [22] (F-actin (red), nucleus (blue), centrosomes (green), pattern (white)). For si*Control* and si*Pkhd1*-treated cells (right), angle distributions of nucleus-to-centrosome vectors are shown to mirror symmetrically (−90° to 90°). Mean vectors of independent experiments (lower left), as calculated from the total range (−180° to 180°), and total mean vector reveal a defect of si*Pkhd1* cells in their ability to correctly place centrosomes towards the migratory front. (*n* =250–310 cells, *n* = 4 independent experiments; *p* = 0.0026; non-parametric test for circular data corresponding to 1-way ANOVA [23]). Size bar, 10 µm. (**B**) In the 2-cell stage, central positioning of centrosomes is critical to initiation of lumen formation and is suppressed by strong peripheral contractility [13]. Using 2-cell stages, relation of relative nuclear to centrosomal distances was determined in untreated cells, on 700 and 1600 µm^2^, and in si*Pkhd1* and si*Control*-treated cells on 700 µm^2^. Graphical illustration is provided in Supplementary Figure S4D. (*n* = 2/5 independent experiments, >130/180 cell pairs per condition for untreated and siRNA-treated cells, respectively; non-parametric *t*-test/Mann–Whitney, *p* < 0.001/0.05, ‡‡‡/*).

*Centrosome positioning is affected in one-cell and two-cell states of FPC-deficient cells.* Relative orientation of organelles reflects the ability of cells to correctly read and respond to environmental cues [22]. On crossbow-shaped micropatterns (of appropriate size), cells rearrange their cytoskeleton and acquire the morphology of a migrating cell with centrosomes (MTOC) and Golgi apparatus positioned in front of the nucleus towards the lamellipodium. Positioning of nucleus and centrosomes of cells on the micropattern is highly reproducible [22]. In our hands, the 1100 µm^2^ crossbow-shaped micropatterns is optimal for MDCKII cells, leading to no detectable differences in shape (aspect ratio) or size of si*Pkhd1* cells and controls (Supplementary Figure S4A–C). However, based on the analysis of nucleus-to-centrosome vectors, positioning of centrosomes is altered in si*Pkhd1* cells and points on the average towards the rear of cells, in contrast to si*Control* cells (Figure 3A). Differences were confirmed using a non-parametric test for circular data.

Initiation of lumen in epithelial spheroids is accompanied by positioning of centrosomes relative to nuclei, which is also an indicator of force distribution between adjacent cells pairs [24]. In high cell confinement, centrosomes are positioned centrally between cells, allowing initiation of apical surface and lumen. In low confinement, cell pairs use larger spreading areas, nuclei are positioned centrally, and centrosomes towards the cell periphery lead to small ratio values of nuclear to centrosomal distances [13] (model, Supplementary Figure S4D). To determine the effect of *Pkhd1* knockdown on centrosome positioning, we studied cell cycle-synchronized single MDCKII cells (cycline-dependent kinase 1 (CDK1)-inhibitor RO-3306, 9 µM) and determined positions of centrosomes and nuclei in their two-cell stages 6 h after inhibitor washout. Cells without siRNA treatment were studied in control experiments to confirm centrosome positioning on 700 µm^2^ as compared to 1600 µm^2^ patterns. Figure 3B reveals that the ratio of normalized nuclear to centrosomal distances is strongly reduced in low cell confinement (1600 µm^2^; for measurement, see supplementary Figure S4E). On 700 µm^2^, reduced FPC expression also leads to a detectable and significant reduction of the nuclear to centrosomal distance ratio by 12% of si*Pkhd1* compared to si*Control*-treated cells.

**Figure 4:**
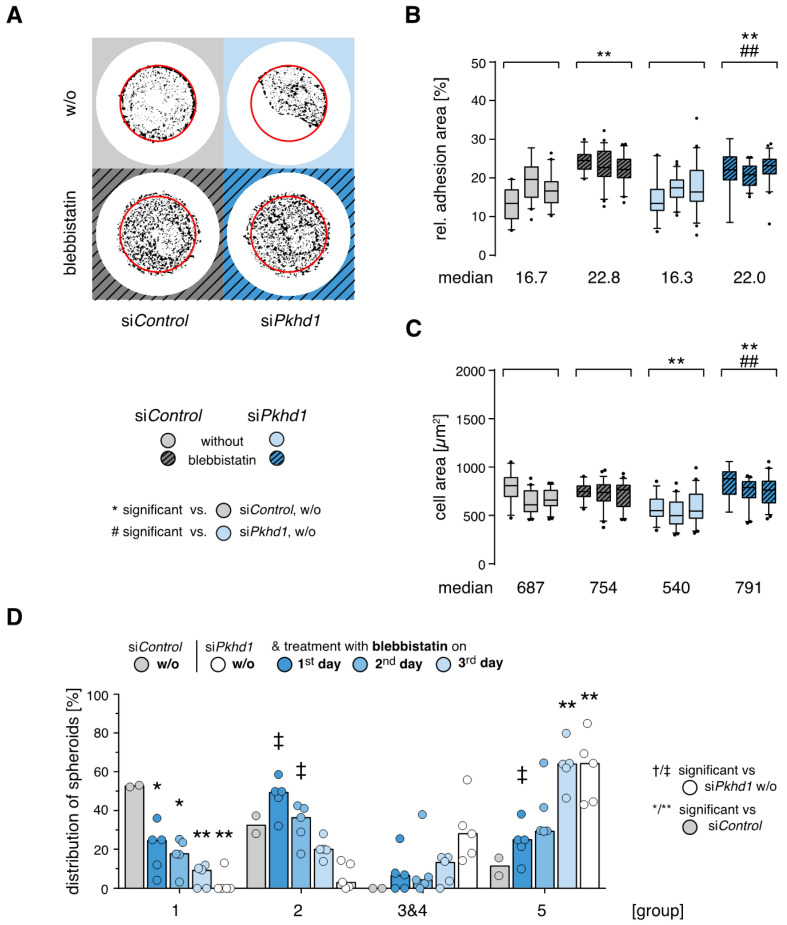
Inhibition of actomyosin contractility reduces cell adhesion and restores spheroid formation of FPC-deficient cells. (**A**) Representative cell shape and adhesion sites of siRNA-treated cells. Three hours after seeding onto 700 µm^2^, cells were treated with myosin II inhibitor, blebbistatin (40 µM), for 1 h. (**B**,**C**) Relative adhesion area and cell spreading of si*Pkhd1* and si*Control* cells on 700 µm^2^ treated with blebbistatin and controls (*n* = 3 independent experiments, >120 cells per condition; two-way ANOVA/Tukey’s, *p* < 0.01, ** or ##.) (**D**) Spheroid formation by siRNA-treated cells was modulated by treatment with blebbistatin for 24 h at different time points after seeding. Inhibition of cell contractility is most effective during the 1st day of spheroid formation, leading to restoration of correct epithelial polarity (groups 1 and 2). (*n* = 2–5 independent experiments, median bars; >140 spheroids per condition; two-way ANOVA/Tukey’s, *p* < 0.05/0.01, */** or †/‡).

*Timed reduction of actomyosin contractility rescues spheroid formation of FPC-deficient cells.* As observed for cells on laminin, adhesion forces and cell contractility triggered by integrin-mediated adhesion signaling strongly affect the outcome of epithelial cell spheroid formation (see above, [13]). After addition of the myosin II inhibitor blebbistatin (40 µM), force transmission of FAs is reduced and cells compensate force balance by enlarging their adhesion areas, building a large number of weak adhesions [18,25]. Blebbistatin treatment is effective in si*Pkhd1* and si*Control*-treated cells, leading to a similar increase of their relative adhesion areas by ≥35% and a loss of restricted cell spreading characteristic for si*Pkhd1*-treated cells under high confinement (700 µm^2^). The impact on the cell area is more pronounced than in si*Control* cells (Figure 4A–C).

Next, blebbistatin treatment was applied in the spheroid assay for 24 h each, on days 1, 2 or 3. Compared to untreated si*Pkhd1* cells, inhibition of cell contractility on day 1 or 2 significantly increased the proportion of correctly polarized spheroids (group 2) and strongly counteracted formation of cell aggregates (group 5) (Figure 4D). Thereby, treatment on days 1 and 2 mostly recovered defects of spheroid formation in FPC-deficient si*Pkhd1* cells as compared to si*Control*-treated cells. With respect to cell aggregates (group 5), significant differences were lost, while there was no full recovery of correctly polarized, complete spheroids (group 1). This may be a consequence of the fact that blebbistatin by reducing actomyosin contractility also inhibits cell division. Thus, in the early stages of spheroid formation, transient reduction of cell contractility can at least in part compensate defective (balance of) adhesion signals in *Pkhd1*-silenced cells and allow normal epithelial morphogenesis.

## 3. Discussion

This study was designed to verify the assumption that mutation or loss of FPC, i.e., the causative events for ARPKD, leads to impaired function of renal collecting duct epithelial cells. Altered binding to and interaction with neighboring cells and extracellular matrix are considered critical aspects of epithelial dysfunction causing damage and/or failure of organs as observed in ARPKD. Therefore, we investigated if the proposed defect in environmental sensing of epithelial cells as well as consequences on epithelial morphogenesis can be demonstrated in vitro by use of a 3D cell culture model.

To this end, we adapted the assay developed in MDCK cells for specifically such purpose by the group of Fernando Martín-Belmonte. This assay was employed by the group to show that cell confinement and spatial control of contractility are critical for centrosome positioning and lumen initiation during epithelial morphogenesis [13]. In particular, starting conditions of the assay applied to single cells provide closely defined and controlled stimuli including matrigel (2.5%) in culture medium, adhesion to one extracellular matrix component and spatial restriction enforced by the micropattern of the adhesion chip. This assay is highly sensitive to variation of ECM protein and loss of confinement, which in our hands was readily reproducible, confirming sensitivity of the morphogenesis assay towards perturbation of the balanced input of stimuli and cellular response. Using transient FPC knockdown in MDCKII cells, we observe a 2/3 reduction in the formation of correctly polarized spheroids (group 1). This robust effect in si*Pkhd1*-treated cells is in the same order as that seen for loss of confinement and clearly indicates altered reaction of FPC-deficient cells to the well-defined set of environmental conditions optimized to induce formation of polarized epithelia by MDCKII cells. With 34% aggregates, si*Pkhd1* cells are evidently not instructed properly to generate polarized epithelia. In addition, we observe 19% inversely polarized cell clusters, which are hardly ever detected in MDCKII controls, whereas there was no change in the number of cells per cluster. Furthermore, the timing of transient reduction (or loss) of FPC function correlates with defective spheroid formation. The initial two-cell stages are critical to the entire process facilitating the fate i.e., polarity and lumen formation, of cell clusters in MDCKII [12,26]. When seeding cells shortly after siRNA treatment, there is no effect of the FPC knockdown. This is consistent with the notion that after two days, when balance of forces between cells and cell polarity are established, disturbance or lack of FPC is not equally relevant to the outcome of the assay than in the initial phase.

An effect of FPC on cell adhesion has been considered previously. In particular, the group of Patricia D. Wilson analyzed aspects of adhesion signaling in conditionally immortalized epithelial cells from normal human fetal collecting tubules and ARPKD cyst-lining epithelia. Their analyses, which are difficult to interpret based on the data provided, suggest altered timing and intensity of focal adhesion kinase and c-Src signaling upon attachment of cells to ECM and enhanced spreading of FPC-deficient cells on collagen [14]. Mai and colleagues observed impaired tubulomorphogenesis of siRNA-silenced murine IMCD cells in a different experimental approach using matrix-based 3D culture. They associate *Pkhd1*-silencing of cells with abnormalities in cell–cell and cell–ECM interactions, the organization of the actin cytoskeleton and in cell proliferation and apoptosis and suggest a link to dysregulated extracellular-regulated kinase and focal adhesion kinase signaling [27]. Puder and colleagues observed in AFM-based experiments on FPC-deficient single cells that control of cell adhesion and cell mechanics are altered [28]. Our approach to assessing control of cell adhesion in si*Pkhd1*-treated MDCKII cells and controls is based on vinculin staining in steady-state adhesions, which provides a reasonable estimate of adhesion forces in integrin-based cell–ECM adhesion sites based on their size and the relative adhesion area [19,21]. In conditions that induce epithelial morphogenesis, we do not observe enhanced cell spreading of si*Pkhd1* cells; however, the equilibrium between actin polymerization-based cell spreading and cell contractility/tension appears to be disturbed [29,30,31]. In high confinement (700 µm^2^), si*Pkhd1* cells in contrast to controls do not even fill the restricted spreading area that is provided, although these cells are not smaller in size, and upon inhibition of myosin II-based contractility, si*Pkhd1* cells display an even larger spreading area than si*Control*-treated MDCKII cells. This finding indicates enhanced contractility in si*Pkhd1* cells and no general problem in actin polymerization. There is no detectable difference in relative adhesion areas between si*Pkhd1* and si*Control*-treated cells, and we also did not pick up alterations in size distribution of individual adhesion sites between both groups. Activation of integrins is a central factor in determining epithelial polarity [32], and correct positioning of centrosomes is essential to apicobasal polarity and a hallmark of epithelial differentiation [33]. Analyzing the relevance of centrosome positioning and distribution of cellular tension for the induction of apical surface and lumen, Rodriguez-Fraticelli and colleagues demonstrated the importance of the spatial distribution of force to cell–ECM and cell–cell adhesions, both of which are linked to the contractile actin cytoskeleton [13,24]. Furthermore, modulation of cell–ECM forces directly affects tension at cell–cell junctions [34]. Thus, we determine two effects in si*Pkhd1* cells which are directly linked to an altered force balance. The positioning of centrosomes, as determined on crossbows (1-cell stage) and by the relative nuclear to centrosomal distance (two-cell stage), is less precise, and most importantly, limitation of actomyosin contraction in the early stage of spheroid formation is critical to the rescue of epithelial morphogenesis.

In summary, correct interpretation of environmental cues is a key feature of epithelial cell function and critical to integrity of epithelia. Upon loss of FPC, sensing of epithelial cells is modified and contributes to progressive defects of epithelial function in ARPKD. Compounds that interfere with enhanced adhesion (signaling) may prove useful to delay progress of the disease. In combination with image analysis based on supervised learning strategies [35], the adhesion chip-based spheroid assay appears suitable for testing of potential therapeutic interventions highly needed for treatment of ARPKD and other inherited forms of PKD.

## 4. Materials and Methods

### 4.1. Cell Culture

Madin-Darby canine kidney cells II (MDCKII; #00062107) were purchased from European Collection of Authenticated Cell Cultures (ECACC) and expanded, and stocks with low passage numbers were stored on liquid nitrogen (cells used between passage 3–10). MDCKII were cultured in minimal essential medium (MEM; Sigma-Aldrich, Taufkirchen, Germany) containing 5% fetal bovine serum (FBS; Biowest, Nuaillé, France), 200 mM L-glutamine (Biochrom; Berlin, Germany) and 1% penicillin/streptomycin (Biochrom) and split every 3–4 days in ratios 1:15 to 1:20.

### 4.2. siRNA and shRNA Treatment (Knockdown)

siRNA protocol: MDCKII cells were split 1:8 two days before treatment. For transfer of siRNA, cell suspensions were treated with Amaxa Cell Line Nucleofector Kit L (VCA-1005; Lonza, Cologne, Germany) according to manufacturer’s manual. In brief, 0.5 × 10^6^ cells were nucleofected with 100 pmol siRNA in nucleofection mix using the Amaxa Nucleofector 2b device (Lonza) and cultured for two days before start of experiments. For analysis of 1-cell polarization and 2-cell states, cells were treated in parallel with 300 nmol of the CETN1-GFP-plasmid to visualize centrosomes [36]. For fibrocystin knockdown (si*Pkhd1*) and control treatment (si*Control*), verified sequences were taken from Zhang et al. [7], si*Pkhd1* (sense) aagcaucaaauccgaguccgu and si*Control* (sense) cguacgcggaauacuucgatt with corresponding antisense RNAs, respectively, and siRNAs purchased from Ambion (Thermo Fisher Scientific Life Technologies, Darmstadt, Germany) as custom Silencer Select.

shRNA adapted protocol: MDCKII cells were split 1:8 two days before treatment. Cells were stably transfected with Tet repressor coding plasmid pcDNA6/TR by reverse transfection using Lipofectamine 2000 (both: Thermo Fisher Scientific, Darmstadt, Germany) and blasticidine selection (5 µg/mL).

shRNA constructs [7] were cloned into the pEmU6proT expression vector (gift from K. Ebnet, University of Muenster, Germany) [17] under control of an U6 promotor and a TetOperator, selection was maintained by G418 (2 mg/mL). shRNA expression was induced by addition of 1 µg/mL doxycycline for 48–72 h to the culture medium. shRNA sequences including BbsI and XbaI restriction site 5′ overhang (5′-3′):

sh*Pkhd1*_1 (fw) tttgaagcatcaaaatccgagtccgtctcgagacggactcggatttgatgcttttttt     (rev) ctagaaaaaaagcatcaaatccgagtccgtctcgagacggactcggattttgatgcttsh*Pkhd1*_2 (fw) tttggcttctatggaaaccctttcactcgagtgaaagggtttccatagaagcttttt     (rev) ctagaaaaagcttctatggaaaccctttcactcgagtgaaagggtttccatagaagcsh*Pkhd1*_3 (fw) tttgcgcagacacccattgtttaccttcaagagaggtaaacaatgggtgtctgttttttt     (rev) ctagaaaaaaacagacacccattgtttacctctcttgaaggtaaacaatgggtgtctgcgsh*Pkhd1*_4 (fw) tttgcgtgccagttcaccgtttggattcaagagatccaaacggtgaactggcattttttt     (rev) ctagaaaaaaatgccagttcaccgtttggatctcttgaatccaaacggtgaactggcacgsh*Control* (fw) tttgaatcgaggtattccgcgtacgctcgagcgtacgcggaatacttcgattttttt     (rev) ctagaaaaaaatcgaagtattccgcgtacgctcgagcgtacgcggaatacctcgatt

Verification of knockdown: Efficiency of knockdown was controlled by quantitative realtime (qRT)-PCR. Two days after nucleofection, mRNA of siRNA treated cells was isolated using RNeasy Mini Kit and RNase free DNase Set (Qiagen, Hilden, Germany). Reverse transcription of 500 ng mRNA (Quanti Tect Reverse Transcription Kit, Qiagen) was followed by qRT-PCR with the Quantifast SYBR Green PCR Kit (Qiagen) on the 7900 HT Fast Real-Time PCR System (Applied Biosystems, Waltham, MA, USA). Relative amounts of *Pkhd1*-mRNA were calculated applying the ΔΔC_T_ method and using the geometric average of three out of four reference genes [37]; glyceraldehyde-3-phosphate dehydrogenase (*Gapdh*) [38], ubiquitin B (*Ubb*) [39], β2- microglobulin (*B2M*) [39] and hypoxanthine-guanine phosphoribosyltransferase (*Hprt*) [40] as detected by canine-specific primer pairs: *Pkhd1* (exon 42/43-44) (fw) tgcactgctagtgggtacag, (rev) ccctcggtgccagaaatact, *Gapdh* (fw) aacatcatccctgcttccac, (rev) gaccacctggtcctcagtgt, *Ubb* (fw) tcttcgtgaaaaccctgacc, (rev) ccttcacattctcgatggtg, *B2M* (fw) tcctcatcctcctcgct, (rev) ttctctgctgggtgtcg, *Hprt* (fw) gcttgctggtgaaaaggac, (rev) ttatagtcaagggcatatcc.

### 4.3. Protein Analysis

MDCKII cells were lysed directly in 65 °C prewarmed 2x Laemmli-Buffer (150 µL/75 cm²) and analyzed using 4–15% gradient gels (4–15% Mini-PROTEAN TGX Precast Protein Gels, Biorad, Hercules, CA, USA) and pre-stained molecular weight markers (PageRuler Prestained Protein Ladder (#26616), 10 to 180 kDa, and HiMark Pre-stained Protein Standard (LC5699), 30 to 460 kDa; Thermo Fisher Scientific). Transfer was performed in Towbin Buffer (0.0375% SDS, without methanol). For immunodetection, rabbit anti-fibrocystin (1:200, C-20, sc-49671; Santa Cruz Biotechnology), mouse anti-vinculin (1:1000, clone hVIN-1; Sigma-Aldrich), and rabbit anti-myosin IIa (1:500, M8064, Sigma-Aldrich) were used. Secondary antibodies, IRDye 800CW goat anti-rabbit IgG (H+L) (1:5000 or 1:10,000) and IRDye 680RD goat anti-mouse IgG (H+L) (1:5000) allowed quantitative fluorescence detection via the Odyssey Fc Imaging System (antisera and detection system, LI-COR Biotechnology, Bad Homburg, Germany). Signal intensities were determined by using Image Studio Software (5.2) and FPC protein normalized using geometric means of vinculin and myosin II signals to adjust protein load.

### 4.4. Spheroid Assay

The spheroid assay was performed on micropatterned chips (CYTOO S.A., Grenoble, France) with disc-shaped micropatterns of 700 and 1600 µm² according to manufacturer’s manual and [13]. In brief, patterns were coated with laminin (20 µg Sigma-Aldrich) or collagen I (20 µg/mL; Sigma-Aldrich). MDCKII cells, 1.5–2 × 10^4^ per well, were seeded in 4-chamber system (CYTOO) allowing four independent conditions per chip and grown in MEM containing 2% FBS, 200 mM L-glutamine and 1% penicillin/streptomycin. After 4 h, half of the medium was replaced by MEM containing, in addition, 5% matrigel (Matrigel Basement Membrane Matrix; Corning, Corning, NY, USA). Within 3 days, single cells adhering to the micropattern divided several times and formed spheroids of 16–20 cells. Optional blebbistatin treatment (40 µM f.c., InSolution™ Blebbistatin; Calbiochem, Merck KGaA, Darmstadt, Germany) was performed for 24 h, on either day 1, 2 or 3 of spheroid growth and terminated by medium change. Spheroids were washed twice with 1 × PBS, fixed with 4% paraformaldehyde in PBS for 15 min, permeabilized with 0.25% Triton-X-100 in PBS for 12 min, blocked for one hour with 5% normal donkey serum (Merck Millipore, Burlington, MA, USA) in PBS, stained with antibodies and dyes (as detailed below) and mounted using Shandon Immu-Mount (Thermo Fisher Scientific). Monoclonal antibodies were mouse anti-gp58 [16,41] (1:200) and mouse anti-gp135-Atto550 [15,42] (1:8000), which were produced by the Antibody Facility (iTUBS, Braunschweig, Germany) and purified/fluorescence-labeled (Hypermol EK, Bielefeld, Germany), and secondary antibody Alexa Fluor 488 donkey anti-mouse IgG (H+L) (1:1000, A-21202; Life Technologies, Carlsbad, CA, USA). Alexa Fluor 660 Phalloidin (A-22285; Life Technologies) was used for F-actin and DAPI (0.25 µg/mL; Sigma-Aldrich) to stain nuclei. Classification of spheroids: Spheroid polarity was determined manually on blinded/masked images by three raters using 4-color z-stacks, stained for basolateral (gp58) and apical (gp135) marker proteins as well as F-actin and nuclei. Raters classified for 3D structure, polarity and lumen. After agreement, treatment groups of images were unmasked and distribution of spheroids within these groups calculated.

### 4.5. 1-Cell Adhesion, Polarization and 2-Cell States

1-cell adhesion: Cells for single-cell adhesion were seeded on micropatterned chips analogous to the spheroid assay. After four hours, cells were fixed as above and stained for adhesion analysis with mouse anti-vinculin (1:1000, clone hVIN-1; Sigma-Aldrich), Alexa Fluor 555 goat anti-mouse IgG (H+L) (1:1000, A-21422; Life Technologies) as well as phalloidin and DAPI (see above). 1-cell polarization: Cells were seeded onto crossbow-shaped micropatterns of 1100 µm^2^, treated as above and stained with mouse anti-γ-tubulin (1:200, clone GTU-88; Sigma-Aldrich), Alexa Fluor 488 donkey anti-mouse IgG (H+L) (1:1000, A-21202; Life Technologies) as well as phalloidin and DAPI (see above). For visualization of micropattern, ECM coating was labeled using a combined of collagen I (see above) and fibrinogen-Atto425 (1 µg/mL; fibrinogen: Sigma-Aldrich; labeling: Hypermol EK). 2-cell states: Forty hours after nucleofection with siRNA, cells were synchronized for ten hours on G2/M-checkpoint using CDK1-Inhibitor RO-3306 (9 µM; Sigma-Aldrich). After eight hours of RO-3306 treatment, cells were seeded on micropatterned chips analogous to spheroid experiments and allowed to adhere in the presence of RO-3306 for another two hours. Thereafter, RO-3306 was removed, and a further six hours later, the bulk of cells had completed cell division. Cells were fixed as described and stained as described for 1-cell polarization.

### 4.6. Image Acquisition

Images were acquired by using the AxioObserver Z1 microscope with the 63× Plan-Apochromat (NA 1.4) oil objective, the AxioCam MRm Rev.3 camera and the software package AxioVision version 4.8.2. (all from Zeiss, Göttingen, Germany). Filter sets used for related staining were (i) filter set 38 HE: gp58/γ-tubulin, Alexa Fluor 488; (ii) filter set 43 HE: gp135, Atto 550 and vinculin, Alexa Fluor 555; (iii) filter set 50: phalloidin, Alexa Fluor 660, (iv) filter set 49: DAPI (filter sets 1–4 from Zeiss) and (v) CFP ET Filterset F46-001(AHF Analysentechnik AG, Tübingen, Germany.

### 4.7. Image Analysis

Cell–ECM Adhesion: Image analysis was performed by using a customized ImageJ macro developed by Marcello Sestu [19]. In brief, in-focus z-plane images of cells stained for vinculin were converted into a mask of the whole cell via edge detection with FeatureJ [43] to define the region of interest (ROI). Background was subtracted with rolling ball algorithm, and within the ROI a binary mask of vinculin-containing adhesion sites was created after intensity-based thresholding. Threshold levels and rolling ball size were adapted to the staining quality of each experiment. Adhesion sites were analyzed excluding areas below 0.1 µm² (Figure S3A). 1-cell polarization: Positions of the pattern, nuclei and centrosomes were detected by application of an intensity threshold to the plane of interest (pattern) or maximum-intensity-projected z-stack of images (nuclei and centrosomes). Positions were calculated relative to the pattern’s center of mass, and subsequently, the nucleus-to-centrosome vectors and the resulting angles to the x-axes determined. Cells <400 µm^2^ covering below 1/3 of the pattern area were excluded from the analysis. 2-cell states: Quantification was performed by using a series of ImageJ macros, adapted from Burute et al. [33]. In brief, nuclear and centrosomal positions were detected by thresholding and size criteria. Based on positional information, distances of nuclei and centrosomes were calculated and the relative nuclear to centrosomal distances determined; see Figure S4E.

### 4.8. Statistical Analysis

To compare parameters from three independent experiments, 2-way ANOVA with Tukey’s multiple comparison was employed, with the exception of treatment in 2-cell states, where a non-parametric *t*-test with Mann–Whitney post hoc analysis was used. All tests were performed using GraphPad Prism version 7 for Windows or MacOS. Analysis of angle distribution was performed in MATLAB (The MathWorks, Inc., Natick, MA, USA) using the “CircStat” Circular Statistics Toolbox [23], wherein a non-parametric test for circular data corresponding to 1-way ANOVA was used.

### 4.9. Data Availability Statement

The datasets generated and analyzed during the current study are available from the corresponding author on reasonable request. ImageJ macros used for image analysis are provided under GNU GPLv3 license on request.

## Supplementary Materials

Supplementary materials can be found at https://www.mdpi.com/1422-0067/21/14/5140/s1, Figure S1: Sensitivity of spheroid formation to confinement, ECM coating and *Pkhd1* silencing by shRNA confirms function of morphogenesis assay, Figure S2: Full-length immunoblot membranes of Figure 1D, showing detection of fibrocystin (FPC), and load controls, vinculin (VCL) and non-muscle myosin IIa (NM IIa), alongside with molecular weight marker bands, Figure S3: Parameter determination of adherent cells and size in solution, Figure S4: Shape and size of cells on crossbow and detection of centrosomes positions in 2-cell stages.

## Abbreviations

3D	three dimensional
AJ	adherens junctions
AFM	atomic force microspcopy
ANOVA	analysis of variance
ARPKD	autosomal recessive polycystic kidney disease
CDK1	cyclin-dependent kinase 1
ECM	extracellular matrix
ESRD	endstage renal disease
IMCD	inner medullary collecting duct
FA	focal adhesions
FPC	fibrocystin/polyductin
MDCKII	Madin-Darby Canine Kidney type II cells
MTOC	microtubule organizing center
PCR	polymerase chain reaction
*Pkhd1*	polycystic kidney and hepatic disease gene 1
ROI	region of interest
sh*Pkhd1*/sh*Control*	name of gene-specific (or control) shRNA constructs
shPool*Pkhd1*	pool of gene-specific shRNA constructs
shRNA	small hairpin RNA
si*Pkhd1*/si*Control*	name of gene-specific (or control) siRNA constructs
siRNA	small interfering RNA
TJ	tight junctions
