# Supplementary material for: Fibrocystin Is Essential to Cellular Control of Adhesion and Epithelial Morphogenesis
Source: Int J Mol Sci. 2020 Jul 20;21(14):5140. doi: 10.3390/ijms21145140 (PMC7404311; doi:10.3390/ijms21145140)
Supplement: Supplementary file 1 [file ijms-21-05140-s001.pdf]

International Journal of
Molecular Sciences

MDPI

Supplementary Materials

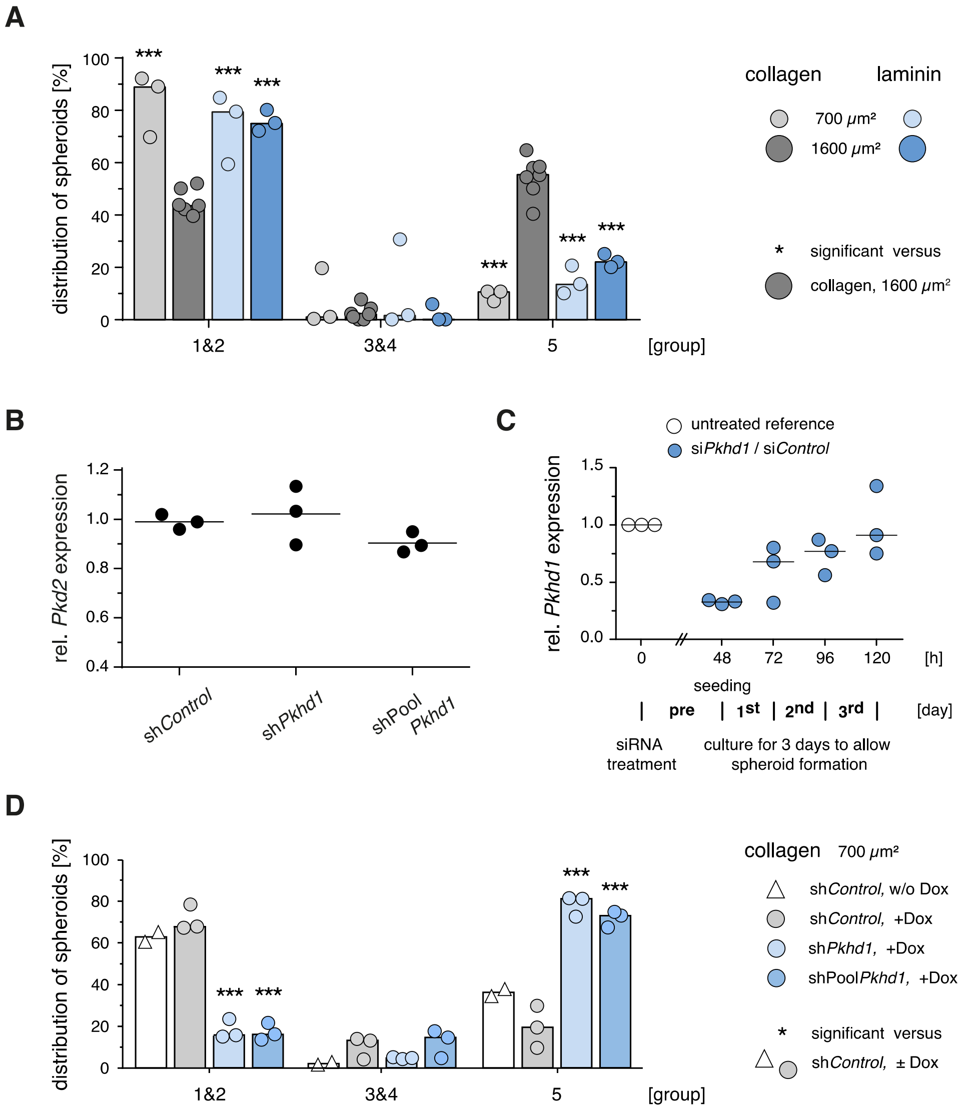

Figure S1. Sensitivity of spheroid formation to confinement, ECM coating and *Pkhd1* silencing by shRNA confirms function of morphogenesis assay. (**A**) Impact of confinement and ECM proteins on epithelial characteristics. Spheroid formation of MDCKII cells on 700 or 1600 µm² pattern coated with collagen or laminin. (n = 3–7 independent experiments, median bars; >150 spheroids per condition; two-way ANOVA/Tukey's, $p < 0.001$, ***). (**B**) *Pkd2* mRNA is not specifically reduced in *Pkhd1* silenced MDCK II cells as compared to control treated cells (n = 3 independent experiments). (**C**) Time course of the MDCKII spheroid assay is shown in its temporal relation to siRNA treatment of cells and si*Pkhd1* knockdown. Knockdown was determined as detailed in the legend of Figure 1C (n = 3 independent experiments). (**D**) Spheroid formation is defective in shRNA-silenced MDCKII TetON-cells [17] with sh*Pkhd1* and shPool*Pkhd1* as compared to sh*Control* expression. Three days after induction of shRNA with doxycycline, cells were seeded on 700 µm² collagen-coated disc-shaped micropattern. Non-induced sh*Control* (-Dox) was included as further control and spheroid formation analyzed for all conditions as described for siRNA, Figure 1E (n = 2–3 independent experiments, median bars; >130 spheroids per condition; two-way ANOVA/Tukey's, $p < 0.001$, ***).

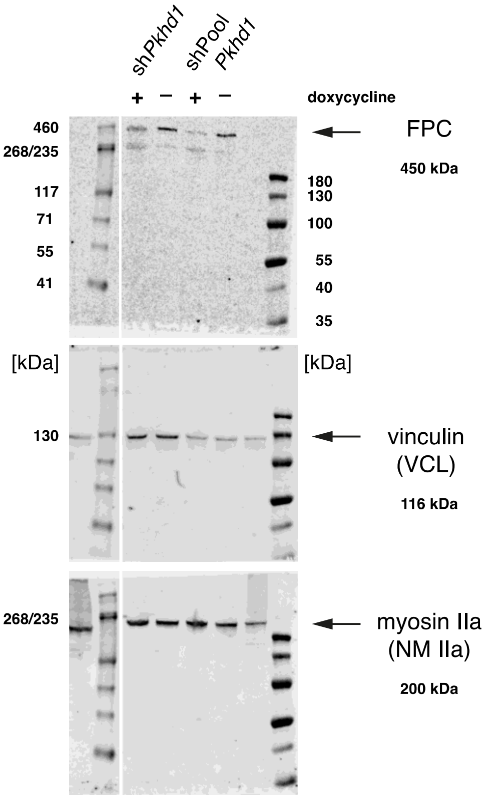

Figure S2. Full-length immunoblot membranes of Figure 1D, showing detection of fibrocystin (FPC), and load controls, vinculin (VCL) and non-muscle myosin IIa (NM IIa), alongside with molecular weight marker bands. Note: protein levels are compared within treatment groups, sh*Pkhd1* and shPool*Pkhd1*, with (+) and without (−) induction of shRNA by doxycycline. There is no impact of shRNA on VCL and NM IIa levels.

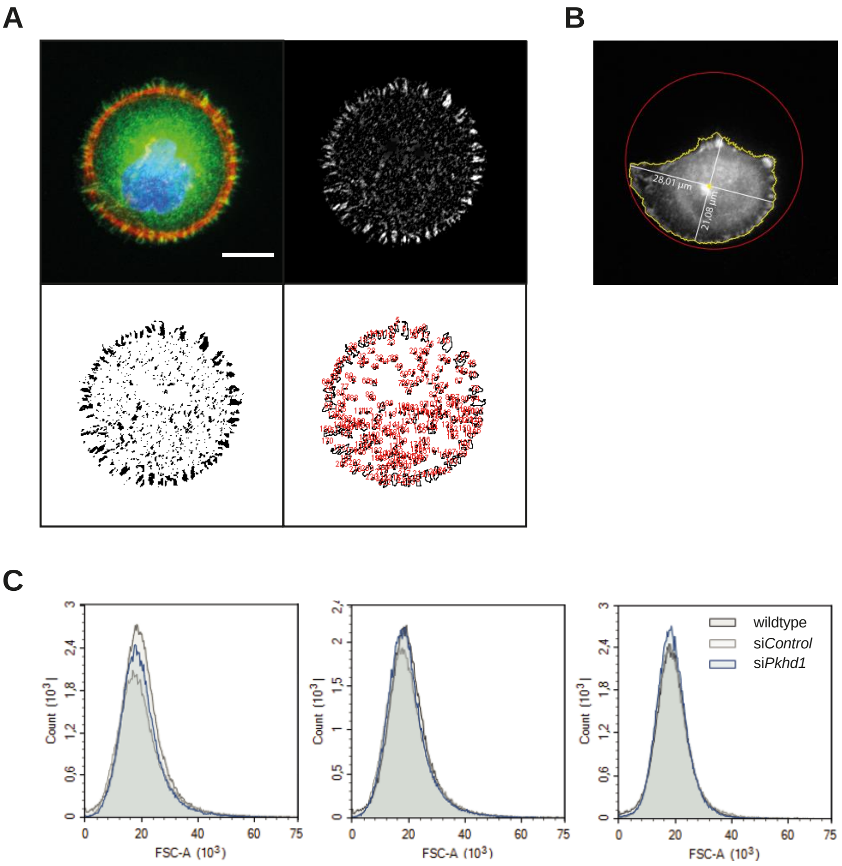

Figure S3. Parameter determination of adherent cells and size in solution. (**A**) Steps illustrate extraction of cell-ECM adhesion sites; (top left) 3-color fluorescence image for vinculin (green), f-actin (red) and nucleus (blue), (top right) background subtracted image (vinculin), (bottom left) binary mask, (bottom right) outlines of extracted adhesion sites (>0.1 μm^2). Outlines (without numbers) are routinely used for visual inspection of adhesion site assignment. Parameters are adjusted to exclude discrepancies between personal observation and automated detection. (**B**) Graph shows measurement of cell length and perpendicular width. (**C**) After siRNA treatment, cell size in suspension is not changed. FACS analysis of MDCKII wildtype, si*Control* and si*Pkhd1*-treated cells showing equal distribution of forward scatter (FSC) parameters for three independent knockdown experiments. Profiles and statistics reveal identical cell size/volume with no detectable shift to higher/lower values of FSC-A.

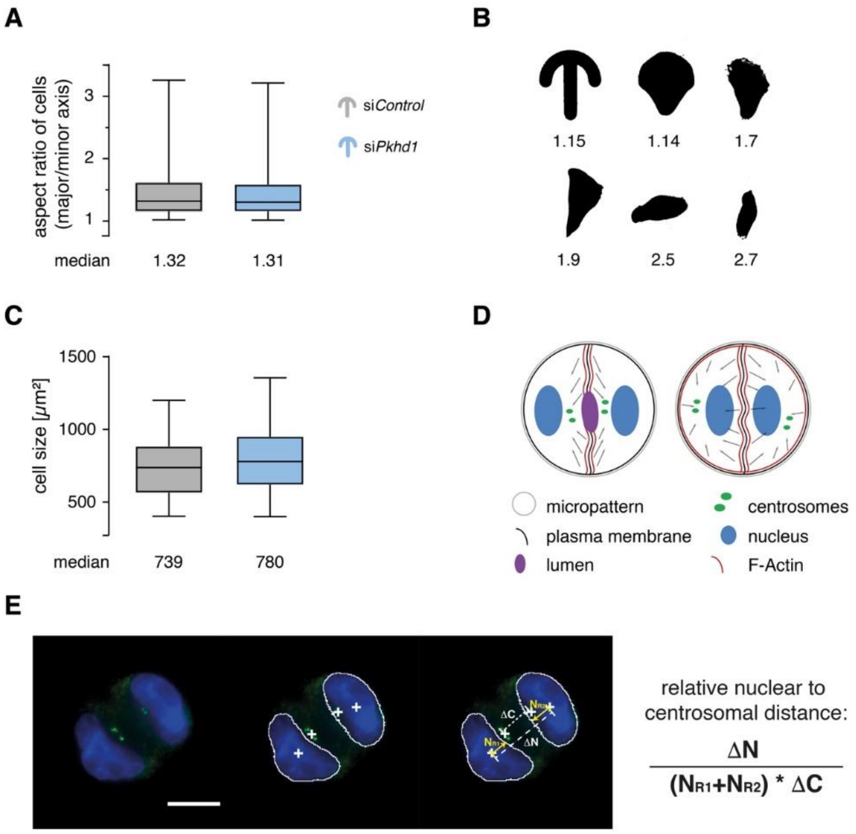

Figure S4. Shape and size of cells on crossbow and detection of centrosomes positions in 2-cell stages. Four hours after seeding, single cells were analyzed on crossbow-shaped micropatterns (1100 µm², CW-M-A, CYTOO) for (**A**) cell geometry, determined as relation of major to minor cell axis, with (**B**) exemplary shapes to illustrate aspect ratios, and (**C**) spreading area, box plot and whiskers 5/95%. (*n* = 250–310 cells, *n* = 4 independent experiments; non-parametric *t*-test/Mann Whitney, not significant) (**D**) Model of centrosome and nuclear positioning in 2-cell states. Under confinement conditions, junctional tension is mostly restricted to cell-cell adhesions favoring induction of apical surface and lumen formation (left). When cell-matrix adhesion is high, peripheral tension pushes nuclei towards the cell-cell junction and inhibits polarization and initiation of lumen (right). Arrows indicate distribution of tension (**E**) Determination of relative nuclear to centrosomal distance; marks indicate centers of mass for nuclei and centrosomes. Size bar, 10 µm.
